# Autologous Peripheral Blood Mononuclear Cells for Limb Salvage in Diabetic Foot Patients with No-Option Critical Limb Ischemia

**DOI:** 10.3390/jcm10102213

**Published:** 2021-05-20

**Authors:** Alessia Scatena, Pasquale Petruzzi, Filippo Maioli, Francesca Lucaroni, Cristina Ambrosone, Giorgio Ventoruzzo, Francesco Liistro, Danilo Tacconi, Marianna Di Filippi, Nico Attempati, Leonardo Palombi, Leonardo Ercolini, Leonardo Bolognese

**Affiliations:** 1 Diabetology Unit, San Donato Hospital Arezzo, Local Health Authorities South East Tuscany, 52100 Arezzo, Italy; marianna.difilippi@uslsudest.toscana.it; 2 Interventional Radiology Unit, San Donato Hospital Arezzo, Local Health Authorities South East Tuscany, 52100 Arezzo, Italy; pasquale.petruzzi@uslsudest.toscana.it (P.P.); nico.attempati@uslsudest.toscana.it (N.A.); 3 Vascular Surgery Unit, San Donato Hospital Arezzo, Local Health Authorities South East Tuscany, 52100 Arezzo, Italy; filippo.maioli@gmail.com (F.M.); giorgio.ventoruzzo@uslsudest.toscana.it (G.V.); leonardo.ercolini@uslsudest.toscana.it (L.E.); 4 Department of Biomedicine and Prevention, University of Rome Tor Vergata, 00133 Roma, Italy; f.lucaroni@gmail.com (F.L.); cristinambrosone@gmail.com (C.A.); leonardo.palombi@gmail.com (L.P.); 5 Interventional Cardiology Unit, San Donato Hospital Arezzo, Local Health Authorities South East Tuscany, 52100 Arezzo, Italy; francesco.liistro@uslsudest.toscana.it (F.L.); leonardo.bolognese@uslsudest.toscana.it (L.B.); 6 Infectious Disease Unit, San Donato Hospital Arezzo, Local Health Authorities South East Tuscany, 52100 Arezzo, Italy; danilo.tacconi@uslsudest.toscana.it

**Keywords:** peripheral blood mononuclear cells, PBMNCs, cell therapy, critical limb ischemia, no-option critical limb ischemia, NO-CLI, diabetic foot, major amputation, amputation-free survival, AFS, wound healing

## Abstract

Peripheral blood mononuclear cells (PBMNCs) are reported to prevent major amputation and healing in no-option critical limb ischemia (NO-CLI). The aim of this study is to evaluate PBMNC treatment in comparison to standard treatment in NO-CLI patients with diabetic foot ulcers (DFUs). The study included 76 NO-CLI patients admitted to our centers because of CLI with DFUs. All patients were treated with the same standard care (control group), but 38 patients were also treated with autologous PBMNC implants. Major amputations, overall mortality, and number of healed patients were evaluated as the primary endpoint. Only 4 out 38 amputations (10.5%) were observed in the PBMNC group, while 15 out of 38 amputations (39.5%) were recorded in the control group (*p* = 0.0037). The Kaplan–Meier curves and the log-rank test results showed a significantly lower amputation rate in the PBMNCs group vs. the control group (*p* = 0.000). At two years follow-up, nearly 80% of the PBMNCs group was still alive vs. only 20% of the control group (*p* = 0.000). In the PBMNC group, 33 patients healed (86.6%) while only one patient healed in the control group (*p* = 0.000). PBMNCs showed a positive clinical outcome at two years follow-up in patients with DFUs and NO-CLI, significantly reducing the amputation rate and improving survival and wound healing. According to our study results, intramuscular and peri-lesional injection of autologous PBMNCs could prevent amputations in NO-CLI diabetic patients.

## 1. Introduction

Critical limb ischemia (CLI) has a high incidence in patients with diabetes and is related with high morbidity and mortality rates [1]. Limb salvage is associated with percutaneous or surgical revascularization, in comparison to the medical treatment in patients with peripheral arterial disease (PAD) and diabetic foot ulcers (DFUs) [2]. However, up to 25% of diabetic patients are not eligible for revascularization as a result of the inability to overcome vessel obstruction and/or for critical general conditions [3,4]. Of the one million annual amputations worldwide, 75% are performed on patients with type 2 diabetes (T2DM) [5]. No-option critical limb ischemia (NO-CLI) remains a strong unmet clinical need: at 1 year follow-up, NO-CLI diabetic patients showed, respectively, lower rates of limb salvage (13.8% vs. 73.4%, *p* < 0.0001), higher rates of amputation (30% vs. 4.5%, *p* = 0.0001), and higher mortality rates (50% vs. 8.9%, *p* < 0.0001) in comparison to revascularizable CLI patients [4]. Autologous cell therapy, and the use of autologous PBMNCs in particular, has arisen as a possible strategy to treat NO-CLI patients as well as diabetic foot patients [6,7,8,9]. Recently, Rigato et al. [10], in a recent meta-analysis of NO-CLI patients, showed that autologous cell therapy had the potential to modify the natural history of intractable CLI. In separate cell type analyses, PBMNCs, but not other cell types, were associated with a significant decrease in amputation and increase in amputation-free survival [10]. Accordingly, Liew et al., in a meta-analysis of 16 randomized trials, showed that PBMNCs lowered the risk of major amputation and significantly increased ulcer healing [11]. The primary mechanism of action of PBMNCs is the induction of therapeutic angiogenesis with collateral vessel formation [12] through the paracrine activities of growth factors, cytokines, and messenger molecules, as well as through exosomes [13]. Moreover, PBMNCs, monocytes/macrophages, and lymphocyte/Treg populations play a key role in tissue regeneration in persistent trophic lesions through inflammatory macrophage M1 polarization to the M2 regenerative phenotype [14,15]. CD14+ monocytes have also been proven to be efficient in patients with diabetes as opposed to the decreased angiogenic activity of CD34+ stem cells [16]. Recent technology improvements have led to the development of less invasive, operator independent, and user-friendly point of care devices based on peripheral blood selective filtration to produce fresh autologous immobilized peripheral blood mononuclear cells, with evidence in term of adequate potency in therapeutic angiogenesis in vitro and in vivo [17]. Promising results were obtained by immobilized PBMNCs produced by point of care selective filtration in different clinical trials [18,19], including in diabetic patients. The aim of this study is to evaluate PBMNC implants in comparison to standard care treatment in NO-CLI patients with DFUs.

## 2. Materials and Methods

This study is a retrospective cohort study approved by the local ethics committee. A cohort of 76 NO-CLI patients with DFUs that were not eligible for revascularization in the first instance according to ESVS ESC 2017 criteria [20], or after multiple revascularization failures, were enrolled and treated with standard medical therapy from January 2014 to February 2019. Data were collected in the hospital’s local database and analyzed retrospectively.

Patients in both groups received the same standard therapy: surgical debridement, local dressings, antiplatelet drugs, pain relief therapy and antibiotics in case of infection signs, and offloading of the affected foot, in accordance with international guidance [21]. Since October 2016, PBMNC filtration technology has been available in our center, and 38 patients were treated with standard care and in addition with autologous PBMNCs.

The inclusion criteria of both cohort groups were: (a) ulcers with inadequate perfusion, as indicated by a transcutaneous oxygen pressure value (TcpO_2_) < 30 mmHg; (b) ulcers with grade I or II or III stage C as defined by the Texas University Classification System [21]; (c) evidence of no run-off pedal vessels, failure after several percutaneous interventions (where re-intervention was no longer possible), or failure after infra-genicular bypass grafting; (d) possibility to save foot support. Exclusion criteria were: (a) lesion site above the tibial–tarsal joint; (b) moderate or severe infection according to the WIFi classification system (The Society of Vascular Surgery—Wound Ischemia and Foot Infection Classification System) [22]; (c) NYHA class IV; (d) anemia (Hb < 8 g/dL); (e) coagulation disorder/thrombocytopenia (PLT < 50,000/µL); or (f) active cancer/leukemia or lymphoma hematological disease.

Both the standard care control group (38 patients) and the PBMNC group (38 patients) received the same diagnostic–therapeutic multidisciplinary approach: diabetes control was maximized by the diabetologist; comprehensive foot assessment was carried out by the nurse, together with the diabetologist, including determination of vibration perception threshold, 10 g monofilament test, and TcpO_2_ measurement; the standard of care includes dressings, off-loading and systemic therapy according to the IWGDF guidelines [23], antibiotic therapy prescribed by infectious disease specialists, and vascular assessment and revascularization procedures performed by cardiologists, vascular surgeons, or interventional radiologists.

Informed consent for participation in the study during the clinical trial was obtained from all subjects.

The concentration of autologous PBMNCs was produced according to the instructions for use by MonoCells–Pall Celeris (Athena) filtration-based point of care device for the rapid preparation of TNC concentrate from 120 mL of anticoagulated blood, for use in human cell therapy applications (now available as Hematrate Blood Filtration System–Cook Regentec). This system is the first point of care device conceived to concentrate an MNC-enriched population of TNCs with high angiogenic potential from PB without apheresis by means of a filtration system. The cell product obtained has been extensively characterized in terms of composition, recovery, and FACS cell population analysis [17]. Briefly, TNCs were enriched 2.97-fold and MNCs were enriched 4.2-fold (average dose implanted = 1.06 ± 0.28 (× 10^8^); the CD34+ progenitor cell subpopulation was enriched by 5.6% ± 4.2% versus peripheral blood with a mean CD34+ cell count of 1.37 × 10^6^. The efficiency of the CD34+ hematopoietic stem cell enrichment of this selective filtration system is comparable with the CD34+ concentration obtained by the use of a point of care device for bone marrow cells (BMAC 2) [17,24]. All procedures were performed in an operating room with anesthesiologic support (propofol and/or peripheral block). After appropriate surgical debridement of the wound bed, multiple perilesional and intramuscular injections of 10 mL PBMNC cell suspensions (0.2–0.3 mL in boluses) were injected along the relevant axis below the knee, at intervals of 1–2 cm and to a mean depth of 1.5–2 cm, using a 21 G needle. This procedure was repeated three times for each patient at intervals of 30–45 days from each other. Foot-sparing surgery in patients treated with PBMNCs was performed at the same time as the final cell implant, and only when the TcpO_2_ value was above 30 mmHg (excluding all patients without foot perfusion improvement). Major amputation was defined as above the ankle amputation. Healing was defined as complete coverage by epithelial regeneration.

**Figure 1:**
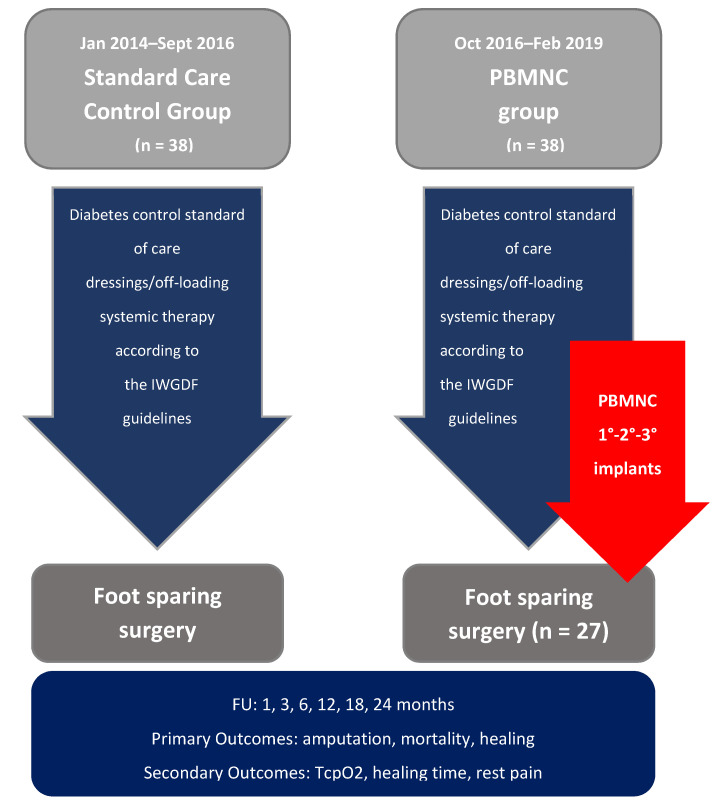
Study flow diagram.

Amputations, risk of death, and healed patients were evaluated as primary outcomes. TcPO_2_ and healing time were evaluated as secondary outcomes. After the first treatment, patients were regularly followed up for two years, with evaluations at 1, 3, 6, 12, 18, and 24 months. See Figure 1 for flow diagram.

### Statistical Analysis

A baseline assessment was carried out to estimate any differences among the standard care control group and the PBMNC group. Due to the small sample size, the evaluation was performed through non-parametric tests (Mann–Whitney U test for independent samples for continuous variables, and Cochrane chi-square test for discrete variables). For patient features and baseline demographics, Bonferroni correction for multiple comparisons was applied and a *p* value equal to 0.003 was considered as the threshold for statistical significance.

A multivariate survival analysis was performed using the Kaplan–Meier survival analysis model by statistical epidemiological software SPSS, version 25. The study size was designed to show a 90% power to identify a proportion of avoided amputations of 70% or greater. Results were considered statistically significant when measures had an estimated error under the 5% threshold: for *p* values <0.05, the null hypothesis was then rejected.

The estimate of relative risk (RR), absolute risk reduction (ARR), relative risk reduction (RRR), and number needed to treat (NNT) was then achieved, with a 95% confidence interval, 5% alpha error, and 20% beta error.

## 3. Results

### 3.1. Patient Features and Baseline Demographics

The study group was composed of 76 patients: 38 patients in the standard care control group and 38 patients in the PBMNC group.

Baseline demographic, clinical, and ulcer characteristics of both groups are reported in Table 1. No significant difference in age, gender, diabetic status (type, duration of disease, and glycated hemoglobin), site of lesion, or number of comorbidities between the two groups was recorded. The prevalence rate of retinopathy was higher in the standard therapy group (X^2^_C_ = 10.077, *p* = 0.002).

### 3.2. Clinical Outcome

The 38 patients treated with PBMNCs showed a significant improvement in all primary outcomes. Kaplan–Meier survival analysis was performed to evaluate amputation-free survival after 1, 3, 6, 12, 18, and 24 months follow-up, comparing the PBMNC and the standard therapy group.

**Figure 2:**
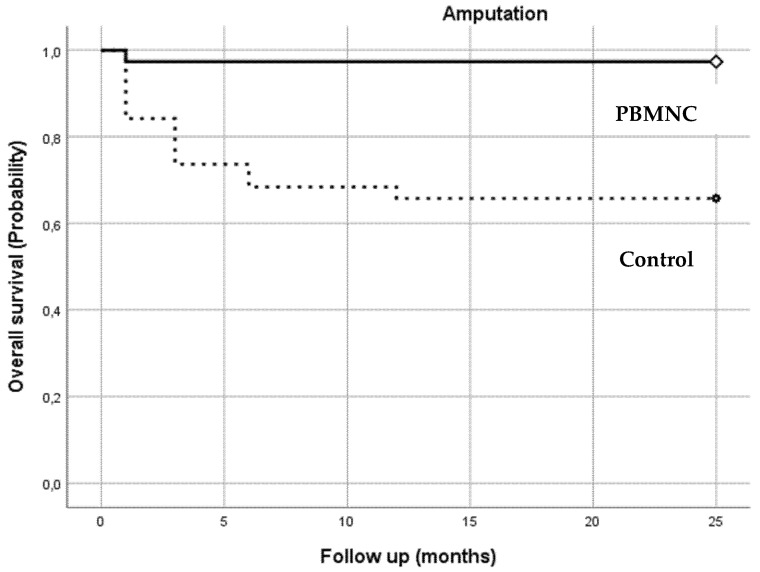
Amputation-free survival: the number of patients alive without amputation in both groups during the follow-up period (1–24 months).

The Kaplan–Meier curves and the log-rank test results showed a significantly lower amputation rate in the PBMNC group (*p* = 0.000; Figure 2) at each point of follow-up. Only 4 out 38 (10.5%) amputations were observed in the PBMNC group, while 15 out of 38 amputations (39.5%) were recorded in the standard care control group (*p* = 0.0037).

**Number at Risk****Months****1****3****6****12****18****24**PBMNC group363434343434Control group322624232323

**Figure 3:**
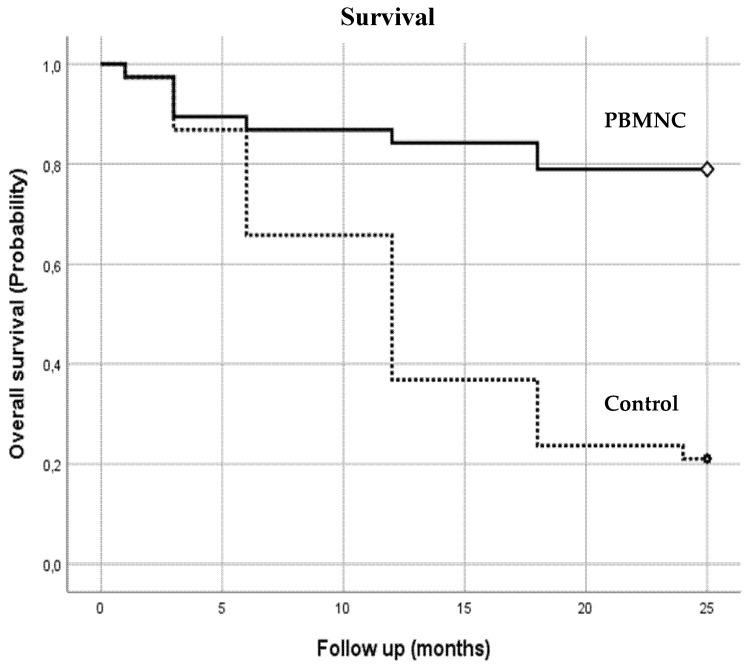
Overall survival: the number of patients alive in both groups during the follow-up period (1–24 months).

Furthermore, mortality risk was significantly lower in the PBMNC group (*p* = 0.000). As illustrated in Figure 3, at the end of the two-year follow up period, nearly 80% of the PBMNC group was still alive (*n* = 30), compared with only 20% of standard therapy group (*n* = 8).

**Number at Risk****Months****1****3****6****12****18****24**PBMNC group373433323030Control group37332716118

**Figure 4:**
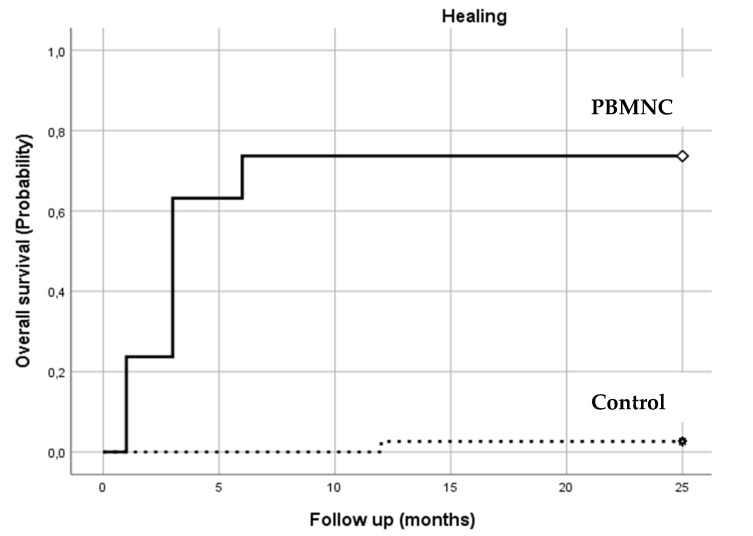
Wound healing: the number of patients healed in both groups during the follow-up period (1–24 months).

As illustrated in Figure 4, almost all the healing events occurred in the PBMNC group (*p* = 0.000). Healing at the two-year follow up occurred in 86.8% (*n* = 33) of the PBMNC group, compared to 2.6% (*n* = 1) of the standard therapy group. Most of the healing events (31 out of 33) in the PBMNC group took place within 6 months of treatment.

**Number at Risk****Months****1****3****6****12****18****24**PBMNC group92531323333Control group000111

Moreover, PBMNC-treated patients showed a decreased risk (RR = 0.11, 95% CI = 0.02–0.52) of amputation compared with the control group, with an absolute risk reduction (ARR) of 0.29 (ARR = 0.29, 95% CI = 0.12–0.46) and a relative risk reduction of 0.85 (RRR = 0.85, 95% CI = 0.36–0.96). The number needed to treat in order to prevent one amputation was 3.45 (NNT = 3.45, 95% CI = 2.19–8.15). Mortality risk within 24 months was higher in the control group (RR = 0.07, 95% CI = 0.02–0.21). Absolute risk reduction was 0.58 (ARR = 0.58, 95% CI = 0.40–0.76) and relative risk reduction was 0.73 (RRR = 0.73, 95% CI = 0.50–0.86), compared with the controls. The number needed to treat to avoid one death was 1.73 (NNT = 1.73, 95% CI = 1.31–2.53).

For the secondary endpoint, TcpO_2_ in the PBMNC group increased by 24 mmHg (median = 24; IQR = 11.5–31.0) at the end of the cell therapy treatment from baseline data (under 25 mmHg is characteristic of CLI). No changes in TcpO_2_ value were observed in the control group during the follow-up period (14 ± 5 mmHg). Healing time was 71.66 ± 42.24 days in the PBMNC group, while only one patient healed in the standard therapy group. No minor or major side effects were observed in the PBMNC group.

## 4. Discussion

Diabetic patients usually suffer from long-segment vascular obstruction, and the predominantly distal vessel disease make these patients poor candidates for revascularization, resulting in continued disease progression, amputation, and death [25,26]. No-option CLI remains a significant unmet medical need, and innovative approaches, such as cell therapy, to induce vascular regeneration and achieve limb salvage are urgently needed. Both mobilized and immobilized PBMNCs have shown promising preliminary results in diabetic patients [6,7,8,9,10,11,18,19]. Our aim was to evaluate PBMNC implant in addition to standard care in NO-CLI patients with DFUs. Despite the limited number of observed patients (*n* = 76) and the scarce sample size (*n* = 38) of the PBMNC patient group, a significant decrease in amputations was observed (4 amputations out of 38 patients) compared to the standard care control group (15 out of 38). Moreover, we observed a low number of deaths (*n* = 8 patients) in the PBMNC group, compared to 30 deaths in the control group. A reduced mortality risk (93% reduction) within two years was recorded for the PBMNC group compared with the standard therapy group.

Furthermore, the additional autologous cell therapy treatment showed a positive impact on healing outcome. Indeed, only one patient treated with traditional therapy healed. The effectiveness of PBMNCs is also highlighted by the assessment of the number needed to treat (NNT) to prevent one additional negative outcome (in our study, death, or amputation). In our study, less than two PBMNC-treated patients should be achieved to avoid one death within 2 years (NNT = 1.73, 95% CI = 1.31–2.53) and 3.45 patients should be treated with PBMNCs to prevent one amputation (NNT = 3.45, 95% CI = 2.19–8.15). The wound healing potency of PBMNCs was previously reported in a meta-analysis, including 16 RCTs and involving 774 CLI patients, where this cell therapy not only significantly lowered the risk of major amputation, but also significantly increased ulcer healing [11].

Death after major amputation in diabetic patients has been well described by Jones et al., who showed that 3 years after below the knee amputation (BKA), 33.3% of patients were dead, and after above the knee amputation (AKA), 71.4% of patients died (*p* < 0.001). At 5 years after BKA, 63.3% of patients were dead, and after AKA, 85.7% of patients were dead (*p* = 0.05) [27]. Persiani et al. [19] reported a 9.4% rate of major amputations in 18 no-option patients with diabetes treated with PBMNCs (produced by the same point of care device used in our study), which is comparable to the 10% amputation rate we observed. The same result was also previously reported in 2009 by Moriya [6], who observed a major amputation rate of 10.5% and a mortality rate of 21.5% at 2-year follow-up in the first published trial on immobilized PBMNC implants in NO-CLI patients. Regarding the standard care control group, our result is similar to a study on 574 NO-CLI patients (of which 70% were diabetic), which reported a 23% major amputation rate and a 31.6% death rate, primarily from cardiovascular disease, after 2 years [28]. Instead, in our study, only 10.5% of patients were amputated and 21.05% died in the PBMNC-treated population at the end of the two-year follow-up. In a previous study on diabetic NO-CLI patients, a 11.1% major amputation rate in the autologous cell therapy group compared with a 50% rate in the control group was observed at 6 months, with no difference between bone marrow cells (BMMNCs) and peripheral blood cells (PBMNCs) [9]. We observed a healing rate of 81.6% and 84.3% at 6 and 12 months compared to a rate of 2.6% in the standard therapy group. Moreover, most patients (31 out of 33) healed after PBMNC treatment within 6 months. The wound healing rate in our study is higher than the rate reported by Dubsky et al. corresponding to 63% and 82% in 31 diabetic NO-CLI patients treated with autologous cell therapy (20 patients treated with BMMNCs produced by a BMAC SmartPrep point of care device and 11 patients treated by G-CSF-mobilized PBMNCs produced by apheresis), respectively) [29]. Interestingly, in the same study, the authors reported a comparable improvement of CLI major amputation with autologous cell therapy compared with repeated PTA, and more effective healing of foot ulcers in the cell therapy group [30].

Recently, Meloni et al. reported a 30% amputation rate and 50% mortality rate for NO-CLI diabetic patients at 1 year follow-up in a retrospective cohort study [4]. Few diseases connote a higher mortality rate: among 22 different types of malignancy, only six have a 5-year mortality rate higher than that of CLI [29]. This tremendously high mortality rate demonstrates the need to identify new therapeutic strategies to reduce major amputation in this fragile population. The 21% mortality rate we observed at two years follow-up in the PBMNC group is a remarkable result compared to the 80% mortality rate of our standard therapy group at two years.

In addition to the positive clinical outcome on amputation mortality and wound healing, the possibility to perform foot-sparing surgery was significantly higher in the PBMNC group (71.05%) compared to the standard therapy group (7.9%), in which the data are similar to a study about this type of surgery in no-option CLI (13%) [31]. TcpO_2_ in the PBMNC group increased by 24 mmHg at the end of cell therapy treatment, while no increase was detected in the control group. A significant increase of TcpO_2_ after PBMNC implants in diabetic patients was previously observed in two clinical trials [9,19]. It was not possible to compare the time to healing between groups because only one patient showed ulcer healing in the control group at the end of the follow-up period.

Pain control is a challenging issue in no-option CLI patients, and it is often only partially controlled by paracetamol and opioids, despite their common side effects such as constipation and drowsiness. In the treated group, pain relief was achieved following the first PBMNC implant, as evaluated by the NRS scale, but data regarding rest pain for the control group were not recorded. Rest pain evaluation with the NRS scale [25] in the PBMNC group showed a mean baseline value of 8.46 +/− 2.01, which decreased to 4.58 +/− 8.39 after the first implant, and ultimately to 2.15 +/− 5.77, allowing the discontinuation of painkillers. Although a direct comparison between the two groups is not possible, rest pain reduction immediately after the first cell implant has also been observed in previous PBMNC clinical trials utilizing PBMNCs generated by a point of care selective filtration system [18,19]. This effect could be partially explained by the fact that macrophages, when polarized in the M2 anti-inflammatory activation state, release powerful natural opioid substances [32]. Interestingly, it has been shown that in streptozotocin-induced diabetic rats, the implantation of peripheral blood mononuclear cell fractions is associated with an improvement in motor nerve conduction velocity (MNCV) due to arteriogenic effects in the sciatic nerve, and that VEGF may contribute to this effect [33]. A reduction in rest pain in CLI patients after PBMNC treatment has been previously reported in clinical trials, as well as in meta-analyses [6,7,8,9,10,11,27].

The frailty of no-option CLI patients and the delicate management of the diabetic foot require that PBMNC therapy, as for the standard therapy, is performed by a multidisciplinary team, which could include care relating to every single feature of the diabetic CLI patient (including the optimization of glycemic control, the reduction of cardiovascular risk factors, the early diagnosis and therapy of infection, pain control, foot surgery, and the early mobilization and rehabilitation of the patient).

Autologous PBMNCs cell therapy could represent an innovative therapeutic strategy to treat these critical patients. PBMNCs offer several advantages over other autologous cellular therapies produced from bone marrow aspirate (such as BM-MNC, or cellular concentrate produced from adipose tissue, such as the stromal vascular fraction (SVF) or micro fragmented adipose tissue), in addition to the obvious non-invasiveness of blood collection. Firstly, PBMNC implants can be repeated easily; a recent randomized controlled trial showed that CLI patients who received four repeated BM-MNC injections versus one single implant show a better pain-free walking distance, suggesting the frequency of implant is superior to cell quantity [34]. Accordingly, Kang et al. confirmed that increasing the injection frequency enhances the survival of the injected bone marrow derived mesenchymal stem cells in a CLI animal model [35]. Secondly, PBMNCs can be easily produced by a point of care selective filtration system intra-operatively and are ready to use in less than 15 min. Thirdly, diabetes heavily impairs bone marrow cell populations, as well adipose tissue cells, both in terms of angiogenic and regenerative ability [36,37]. In the recent MOBILE randomized double-blind study on 152 no-option CLI patients at Rutherford stage 4 or 5 treated with BM-MNC or placebo, the 2-year post-hoc analysis showed that while BM-MNCs did provide a significant benefit for patients without diabetes at Rutherford stage 4, it did not provide any benefit for patients with diabetes and/or those at Rutherford stage 5, suggesting a negative impact of diabetes on cell therapy with BM-MNC for CLI [38]. Recently, the SCELTA trial suggested the “non-inferiority” of non-mobilized PBMNCs compared to BM-MNCs [8]. Given the current absence of evidence of the superiority of bone marrow versus peripheral blood cells, the advantage of peripheral blood as a cell source is the avoidance of bone marrow harvesting disadvantages such local pain, hematomas, and anemia, as well as a longer surgical procedure [10]. Dong et al. showed that there are no differences in amputation-free survival in patients treated with purified CD34+ or PBMNCs in a randomized trial [39]. Diabetes impairs the angiogenic capacity of human adipose-derived stem cells, mainly by the reduction of the CD271 + subpopulation [40]. So far, there are few studies on the use of adipose tissue cell concentrates for CLI in diabetic patients, and adipose tissue concentrates have not been included in meta-analyses [41,42]. A recent study observed a dysfunction in mesenchymal stem cells from the adipose tissue of diabetic patients, probably due to oxidative stress and autophagy, suggesting a limit to their therapeutic use [43]. On the contrary, adipose tissue concentrate has been shown to be safe and efficient to treat chronic venous ulcers [44].

Although this study is exposed to several potential biases as a result of its nonrandomization and the relatively small sample size, the intramuscular and peri-lesional injection of autologous PBMNCs showed very encouraging results without any adverse effects on all primary end points evaluated (amputation, death, and wound healing) in the two-year follow-up period.

## 5. Conclusions

In the last few years, a huge number of papers studying the mechanism of action of PBMNCs have been published, both on their characteristic angiogenic potency and on their regenerative and immunomodulatory capacity through the polarization of macrophages [12,43,44]. The new concept of the immune-centric revolution shifts the focus from stem cells to immune cells, particularly monocytes/macrophages and lymphocyte-based cell therapy, in regenerative medicine [44]. In our study, autologous PBMNCs, produced easily in the operating room by a dedicated selective filtration point of care device, seem to be a very promising therapy, with the potential to modify the natural history of intractable CLI and diabetic foot in terms of major amputation and overall survival rates. PBMNC therapy opens a new frontier in the management of these critical patients.

## Figures and Tables

**Table 1:** Patient features and baseline demographics.

	PBMNC Group	Control Group	Statistical Test	*p* Value
Age	77.00 ± 6.72	77.58 ± 10.73	U = 664.500	*p* = 0.55
Gender	26M (68.4%) 12F (31.6%)	26M (68.4%)12F (31.6%)	X^2^_C_ = 0.000	*p* = 1.000
Type of diabetes	Type 1 = 3 (7.9%) Type 2 = 35 (92.1%)	Type 1 = 1 (2.6%) Type 2 = 37 (97.4%)	X^2^_C_ = 1.056	*p* = 0.304
Duration of diabetes	16.45 ± 8.96	18.63 ± 8.60	U = 621.000	*p* = 0.291
Site of lesion	Forefoot (78.9%); hindfoot (21.1%)	Forefoot (73.7%); hindfoot (26.3%)	X^2^_C_ = 0.291	*p* = 0.589
HbA1c %	7.48 ± 0.69 (58 mmol/L)	7.62 ± 0.77 (60 mmol/L)	U = 622.000	*p* = 0.389
Rheumatologic disease	12 (31.6%)	9 (23.7%)	X^2^_C_ = 0.592	*p* = 0.442
Cardiopathy	23 (60.5%)	27 (71.1%)	X^2^_C_ = 0.935	*p* = 0.333
Stroke/TIA	8 (21.1%)	17 (44.7%)	X^2^_C_ = 4.828	*p* = 0.028
Retinopathy	8 (21.1%)	21 (55.3%)	X^2^_C_ = 10.077	*p* = 0.002 *
Neuropathy	26 (68.4%)	31 (81.6%)	X^2^_C_ = 1.754	*p* = 0.185
Wound extension (Texas University Classification)	2C = 9 (23.7%) 3C = 29 (76.3%)	2C = 5 (13.2%) 3C = 33 (86.8%)	X^2^_C_ = 1.401	*p* = 0.237
WIFi	W1I3Fi0 = 10 (26.3%) W3I3Fi0 = 28 (73.7%)	W1I3Fi0 = 4 (10.5%) W3I3Fi0 = 34 (89.5%)	X^2^_C_ = 3.152	*p* = 0.076
TcpO_2_	11.59 ± 5.2	14.05 ± 5	U = 581.500	*p* = 0.196
Renal failure	21 (55.3%)	19 (50.0%)	X^2^_C_ = 0.211	*p* = 0.646
Angioplasty Failure	30 (78.9%)	21 (55.3%)	X^2^_C_ = 4.828	*p* = 0.028 *
Not feasible	8 (21.1%)	15 (40.5%)	X^2^_C_ = 3.348	*p* = 0.067
Bypass occlusion	5 (13.2%)	4 (10.8%)	X^2^_C_ = 0.098	*p* = 0.754
Tibial/pedal absence	23 (67.6%)	29 (76.3%)	X^2^_C_ = 0.67	*p* = 0.412
Calcification	24 (75.0%)	34 (89.5%)	X^2^_C_ = 2.56	*p* = 0.109

Legend: * *p* < 0.003 (*p* value with Bonferroni correction). HbA1c % = glycated hemoglobin; TIA = transient ischemic attack.

## Data Availability

The data presented in this study are available on request from the corresponding author. The data are not publicly available due to privacy reason.

## Clinical Trial Registration

NCT04255004.
